# Research on preparation of phosphate-modified animal glue binder for foundry use

**DOI:** 10.1098/rsos.171795

**Published:** 2018-03-07

**Authors:** Tian-Shu Wang, Wei-Hua Liu, Ying-Min Li

**Affiliations:** School of Materials Science and Engineering, Shenyang University of Technology, Shenyang 110870, People's Republic of China

**Keywords:** animal glue, anhydrous sodium carbonate, composite phosphate, foundry binder

## Abstract

In this paper, three phosphates were used as modifiers to modify animal glue binder. The structural characteristics and thermal properties of animal glue binder treated with phosphates were studied by Fourier transform-infrared spectroscopy, gel permeation chromatography and derivative thermogravimetric analysis. The results showed that the modified animal glue binder had better sand tensile strength and lower viscosity than untreated animal glue binder. The best modification process was as follows: the optimal amount of sodium carbonate was 4 wt% to animal glue; the optimal weight ratio of the modifiers was sodium pyrophosphate : sodium tripolyphosphate : sodium hexametaphosphate : animal glue = 3 : 3 : 4 : 100, and the optimal reaction should be performed at 80°C for a reaction time of 120 min. A final tensile strength of approximately 3.20 MPa was achieved and the viscosity value was approximately 880 mPa s.

## 1. Introduction

Natural foundry binders derived from clay and starch reached their peak use in the early 1960s [1]. However, they were subsequently supplanted by petroleum-based synthetic resins. It is commonly considered that after pouring, the resin sand may exhibit some shortcomings such as causing environmental pollution and waste of natural resources [2].

The animal glue binder system's raw materials are derived from renewable natural sources [3]. They are non-toxic and environmentally benign. These raw materials are purified and processed to give the binder the characteristics that allow it to be used as a sand binder. Most animal glue binders are a combination of various types of polypeptide molecules, or long chains of amino acids. The binder, which is a dry, fine, slightly tan powder, is not flammable or reactive [4].

In 1996, researchers at General Motors developed a mixture of proteins of various molecular weights intimately mixed with a metal oxide catalyst to enhance thermal degradation. At that time, General Motors was issued a patent for the new GMBOND sand binder, which was licensed for production [5].

In 1998, the researchers from Harbin Institute of Technology used the animal glue as sand core binder and the tensile strength of samples was 2 MPa [6]. Also, they performed systematic research on the animal glue sand casting process and regeneration method of the used core sand [7].

Furthermore, research on animal glue binders during the past 10 years has been largely directed towards viscosity, primarily because of its high viscosity and poor fluidity at room temperature. To overcome this problem, the research on animal glue binders was mainly based on acid hydrolysis (or alkaline hydrolysis) and organic modifiers.

Research from Shenyang University of Technology introduced a new modified animal glue foundry binder, which was prepared with acrylic acid and glucose, with a higher tensile strength and lower surface tension. This research found that the structure of the binder was changed and it was a liquid at normal temperature [8]. Fox *et al.* [9] provided a new core binder, which was a combination of collagen and lithium/potassium silicate or sodium silicate; a foundry binder that possesses favourable physical and mechanical properties was formed. Moreover, the novel collagen plus silicate binders compared favourably to conventional modified sodium silicate and phenolic urethane. Miao *et al.* [10] developed a type of bone glue adhesive by modification with 1.5 g NaOH and 0.5 g epichlorohydrin. They found that NaOH could make the binder molecules smaller, leading to a decrease in viscosity. A mixed acid was used by Su *et al.* [11] to hydrolyse animal bone binder, and glutaraldehyde was used in the preparation of the binder, which has a lower freezing point and viscosity than an unmodified binder.

There are few research works on the modification of animal glue binder by means of an inorganic modifier. The purpose of this paper is to research the characteristics of the modified animal glue binder system using the salt of a strong alkali and weak acid and some inorganic salt modifiers. The new modified animal binder was characterized and analysed by means of Fourier-transform infrared spectroscopy (FTIR), gel permeation chromatography (GPC) and derivative thermogravimetric analysis (DTG).

## 2. Experimental procedures

### 2.1. Materials

The animal bone glue, which was made from animal fur and bone, consisting of yellow solid granules with a pH of 6.8, was from Liaoning Xinxing Co., Ltd, China. The sand ‘ZGS-50/100(60)’, with a moisture content less than 0.3%, was from Dalian, China. Anhydrous sodium carbonate, sodium pyrophosphate, sodium hexametaphosphate and sodium tripolyphosphate were from Sinopharm Chemical Reagent Co., Ltd, China.

### 2.2. Preparation of animal glue binder

Water (140 g), 100 g of animal glue and a certain amount of hydrolysis catalyst were mixed in a 1000 ml three-neck flask (24/29 bore diameter) for 40 min. Then, the sodium pyrophosphate, sodium hexametaphosphate and sodium tripolyphosphate were added.

After 80–180 min of reaction an anticoagulant was added, and the binder was stirred for another 30 min and cooled to room temperature. Finally, a brown liquid binder was obtained. An animal glue binder with no modifier was also prepared for comparison. The binders were kept at room temperature for three months.

### 2.3. Preparation of sand sample

At first, 1000 g of standard sand was mixed with 30 g binder for 120 s. The sand mixture was used to prepare the standard ‘8’ sample with a hammer machine. Then the samples were baked at approximately 180°C for 20 min and cured in an oven. Finally, the sand samples were taken out and cooled to room temperature for the test.

### 2.4. Determination of tensile strength of samples

The tensile strength of the prepared samples was measured using an ‘SWY’ testing machine.

A sample was put on the testing machine and gradually loaded until it was broken, and its tensile strength value could be read from the instrument. The final result was the average of five samples.

### 2.5. Measurement of binder viscosity

The viscosity of the binder at room temperature was measured by an ‘NDJ-1’ viscosity meter. Cooled to room temperature, 300 g of animal glue binder was placed in a beaker and rotated for 1 min at a speed of 30 r min^−1^ (3^#^ rotor was used). The viscosity could be read directly from the device. The test was repeated three times to obtain an average value.

### 2.6. Orthogonal test design

The orthogonal test with three factors and three levels was designed to screen the extraction conditions, including sodium pyrophosphate, sodium tripolyphosphate and sodium hexametaphosphate. The parameters of the L_9_ (3^3^) orthogonal test are presented in table 1. The tensile strength of a sand sample was tested as an important property index of the binder.

**Table 1.** Orthogonal test with three factors and three levels L_9_ (3^3^) for the modified animal glue binder.

	orthogonal test factor		
treatment	sodium pyrophosphate (g)	sodium tripolyphosphate (g)	sodium hexametaphosphate (g)
T1	1.0	1.5	3.0
T2	1.0	3.0	4.0
T3	1.0	4.5	5.0
T4	3.0	1.5	4.0
T5	3.0	3.0	5.0
T6	3.0	4.5	3.0
T7	5.0	1.5	5.0
T8	5.0	3.0	3.0
T9	5.0	4.5	4.0

### 2.7. Fourier-transform infrared spectroscopy

The FTIR spectra of samples were obtained on an IR Prestige-21 infrared spectrometer made in the USA. All the animal glue samples were cured in an oven at 40°C for 10 h to ensure that the water had entirely evaporated. Then, the samples were ground into very fine powder. The powder samples were mixed with KBr. At that point, the mixture was compressed into pills that could be placed into the spectrometer for FTIR scans.

### 2.8. Derivative thermogravimetric analysis

The DTG analysis was performed using a NETZSCH TGA spectrometer, type TG-209, made in Germany. The experimental conditions were as follows: heat rate, 5°C min^−1^; temperature range, 0–800°C; and nitrogen flow rate, 60 ml min^−1^.

### 2.9. Gel permeation chromatography

The GPC analysis was carried out using a Viscotek TDA305max multi-detector GPC, made in England. The experimental conditions were: 50 mM PB + 50 mM NaCl (pH = 6.8–7.0), SRT SEC-300 column, 0.5 ml min^−1^ flow rate, 100 µl injection volume, 25°C column temperature and 25°C test temperature. The standard product was blood serum albumin (BSA) purchased from Sinopharm Chemical Reagent Co., Ltd, China.

## 3. Results and discussion

### 3.1. Determination of hydrolysis process

Animal glue is a macromolecular compound and it easily agglomerates at room temperature, so it is difficult to be used in a foundry directly. By catalytic decomposition, the peptide molecular structure of the animal glue can be broken down to a smaller molecular structure, which is liquid at room temperature and apt for chemical reaction.

The effects of different hydrolysis catalysts, added under the same conditions, on the tensile strength of casting sand was as shown in table 2.

**Table 2.** Tensile strength of moulding sand with different catalysts.

catalyst	viscosity (mPa s)	tensile strength (MPa)
no catalyst	2500	1.50
hydrochloric acid	1800	1.71
citric acid	1500	1.45
anhydrous sodium carbonate	1100	2.15
potassium hydroxide	1350	2.00
calcium hydroxide	1550	2.00

As shown in table 2, after the hydrolysis with anhydrous sodium carbonate, the tensile strength of the samples was the highest and the viscosity of the binder was the lowest, because the carbonate ion of anhydrous sodium carbonate can be ionized in aqueous solution. Moreover, it can react with hydrogen ions from water to form bicarbonate ions, which leads to the decrease in hydrogen ions in the solution. Furthermore, the weakly alkaline solution is beneficial to promoting the hydrolysis of animal glue. So, in this paper, anhydrous sodium carbonate is used as the hydrolysis catalyst for animal glue.

**Figure 1.**
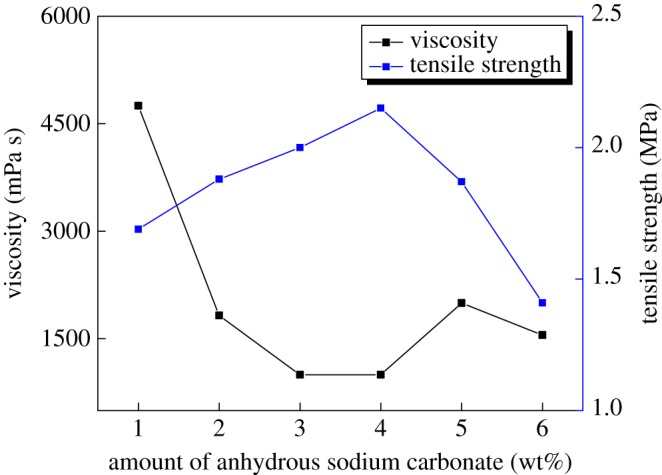
Effect of anhydrous sodium carbonate on the animal glue binder.

The influence of an added amount of anhydrous sodium carbonate on the animal glue binder was tested and is shown in figure 1.

As shown in figure 1, with an increase in added anhydrous sodium carbonate, up to 4%, the tensile strength of the sand sample increased, and meanwhile the viscosity of the animal glue decreased notably. When the added amount of anhydrous sodium carbonate was 4%, the highest point of tensile strength of sand and the lowest viscosity of the binder were demonstrated in testing. When there is a higher level of anhydrous sodium carbonate in the reaction system, the tensile strength begins to decrease and the viscosity significantly increases. The lower viscosity of the binder, with better fluidity, is beneficial for adherence to the sand surface and increasing the binder strength.

### 3.2. Design of composite modification and orthogonal test

Animal glue is a macromolecular compound that consists of many multiple amino acid molecules linked by peptide bonds. After hydrolysis, some structures of peptide bonds may be decomposed to amidogens and carboxyls. Some of the modifiers were selected and some cross-linking reactions occurred.

A cross-linking reaction can take place between the hydroxyl radical (−OH) of animal glue and the phosphate radical (PO_4_^3−^) of phosphate modifiers (sodium pyrophosphate, sodium hexametaphosphate, sodium tripolyphosphate), generating P−O–C chemical bonds. The modification of phosphate can not only reduce the gel properties of animal glue molecules but also improve the emulsifying properties, foaming properties and stability of the binder, thereby enhancing their adhesive properties and increasing bond strength.

**Figure 2.**
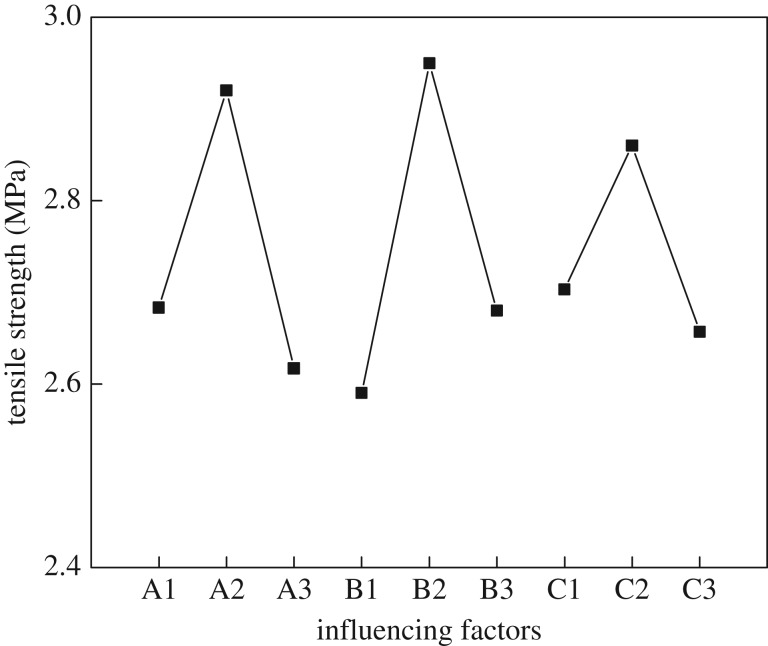
The range analysis of the modifiers.

To evaluate the effect of three factors on the performance of animal glue binder and to find the main factors and optimization scheme, a range analysis of the orthogonal experiment was carried out. The results are presented in table 3 and figure 2.

**Table 3.** Result and analysis of the orthogonal experiment.

	orthogonal test factor				
treatment	sodium pyrophosphate (g)	sodium tripolyphosphate (g)	sodium hexametaphosphate (g)	tensile strength (MPa)	viscosity (mPa s)
T1	1.0	1.5	3.0	2.45	1775
T2	1.0	3.0	4.0	2.96	1075
T3	1.0	4.5	5.0	2.64	1125
T4	3.0	1.5	4.0	2.99	1000
T5	3.0	3.0	5.0	3.00	800
T6	3.0	4.5	3.0	2.77	950
T7	5.0	1.5	5.0	2.33	1375
T8	5.0	3.0	3.0	2.89	1025
T9	5.0	4.5	4.0	2.63	1250
*k*_1_	2.683	2.590	2.703		
*k*_2_	2.920	2.950	2.860		
*k*_3_	2.617	2.680	2.657		
*R*	0.303	0.360	0.203

As can be seen in table 3 and figure 2, the sequence of modifiers affecting the tensile strength is sodium tripolyphosphate, sodium pyrophosphate and sodium hexametaphosphate. Under T5 treatment (sodium pyrophosphate : sodium tripolyphosphate : sodium hexametaphosphate : animal glue = 3 : 3 : 5 : 100), the highest tensile strength of all treatments was observed. However, according to the tensile strength average value (*K*) of each modifier, 3.0 g sodium pyrophosphate, 3.0 g sodium tripolyphosphate and 4.0 g sodium hexametaphosphate were selected to be further tested; compared to T5, this showed better effects not only on tensile strength (3.20 MPa) but also on viscosity (880 mPa s).

Thus, the best ratio of the modifiers by weight was determined to be sodium pyrophosphate : sodium tripolyphosphate : sodium hexametaphosphate : animal glue = 3 : 3 : 4 : 100.

### 3.3. Optimization of modification process

**Figure 3.**
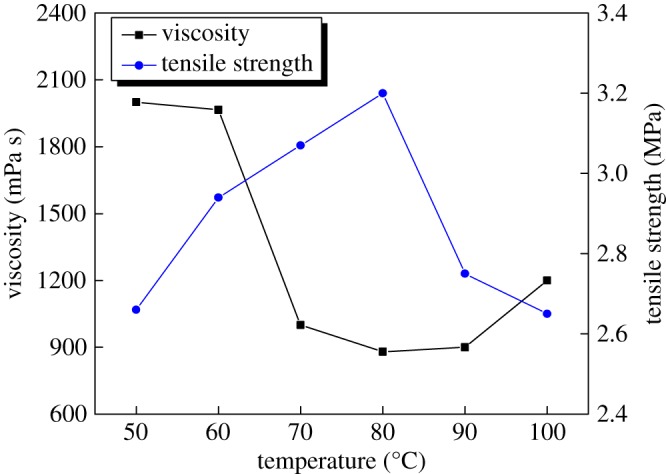
Effect of modification reaction temperature on tensile strength and viscosity of the animal glue binder.

While other conditions remain the same, the influence of modification reaction temperature on tensile strength and viscosity of animal glue binder is shown as figure 3.

As can be seen in figure 3, the tensile strength of the samples increased at first with the increase of the reaction temperature, reached a peak at 80°C and then declined sharply. The viscosity of animal glue binder rapidly decreased to a minimum value at 80°C, and then increased slowly. So the optimal temperature for modification reaction temperature was shown to be 80°C.

**Figure 4.**
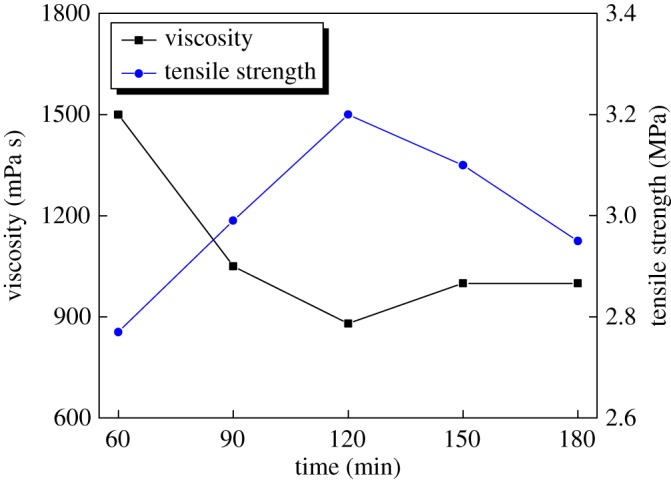
Effect of modification reaction time on tensile strength and viscosity of the animal glue binder.

The influence of modification time on the binder was tested and is shown in figure 4.

With the extension of the modification reaction time, the tensile strength of the binder increased and reached a peak strength value at 120 min, while the viscosity of the binder was just the lowest. So the optimal reaction time was 120 min.

### 3.4. Characterization results of animal glue binder

**Figure 5.**
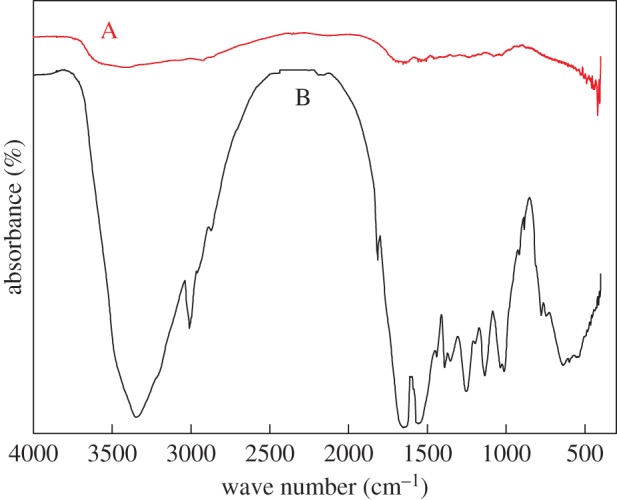
IR spectra of the animal glue binder: Line A, unmodified animal glue binder; Line B, modified animal glue binder.

The IR spectra were analysed for the unmodified animal glue binder and the treated phosphate-modified binder. The results are shown in figure 5.

As can be seen in figure 5, the peak at the range of 3500–3000 cm^−1^ was an absorption peak containing the –OH and the –NH stretching vibration absorption peaks. In the animal glue binder cross-linked with phosphate, the absorption was weaker than in the unmodified binder, which might be a result of the reaction of O–H groups of the animal glue binder. The wide peak near 633 cm^−1^ was an O–P–O bending vibration absorption peak. The O–P–O stretching vibration peak was seen at 1023 cm^−1^. The right absorption acromion near 1248 cm^−1^ was a P–O–C bond stretching vibration; the absorption at 1346 cm^−1^ was assigned to P = O stretching. Moreover, bending vibration of P–O–P was not found at 800–750 cm^−1^. This indicated that there was a cross-linking reaction between the phosphate of the modifiers and the hydroxyl of the animal glue, rather than a polyphosphate in combination with the animal glue binder.

**Figure 6.**
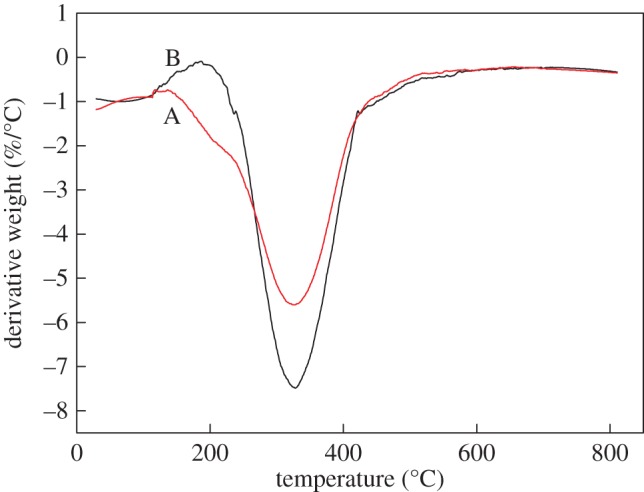
DTG of the animal glue binder: Line A, unmodified animal glue binder; Line B, modified animal glue binder.

Figure 6 presents the DTG of unmodified animal glue and phosphate-modified binder.

As can be seen from figure 6, it was found that the thermal degradation of animal glue binder is identified to be in two steps, and the maximum decomposition temperature of the modified binder (approx. 330°C) is a little higher than that of the unmodified binder. The first stage occurs before 150°C, which is mainly caused by loss of water by evaporation from the binder; the second stage occurs in the range of 300–500°C, during which the peptide bonds break, causing gradual decomposition into peptides and amino acids; the amino acid residues are destroyed by deamination and dehydration.

Table 4 shows the GPC results of binders with or without phosphate modifiers.

**Table 4.** Distribution of molecular weight of animal glue binder.

	weight-average molecular weight (Mw g mol^−1^)	number-average molecular weight (Mn g mol^−1^)	polydispersity
unmodified animal glue	720 810	267 960	2.68
modified animal glue	359 341	289 963	1.24

As can be seen from table 4, the number-average molecular weight (Mn) of the phosphate-modified animal glue binder was higher than that of the unmodified binder, but the distribution was narrower. After modification, the molecular weight of the binder was increased, indicating that the molecules of the animal glue and the phosphate are grafted and cross-linked to form a larger molecule, and the smaller molecular component in the binder made the molecular weight distribution narrower.

## 4. Conclusion

A new animal glue binder which is liquid at room temperature was prepared by hydrolysis reaction and modification. It can provide the higher tensile strength of sand cores and the low viscosity of a liquid animal glue binder. According to the experimental results, the following conclusions can be made:

(1) Anhydrous sodium carbonate can be used as the catalyst for the hydrolysis reaction; the optimal amount is 4 wt% of the animal glue.
(2) The optimal weight ratio of the modifier is sodium pyrophosphate : sodium tripolyphosphate : sodium hexametaphosphate : animal glue = 3 : 3 : 4 : 100, the optimal temperature of the modification reaction is 80°C and the reaction time is 120 min.
(3) The IR spectra proved that the structure of the binder had been changed after the modification process. IR analysis showed that a cross-linking reaction occurred. The DTG results showed that the thermal stability of the modified binder was improved. The GPC results showed that the number-average molecular weight (Mn) of the phosphate-modified binder was higher and its distribution was narrower.

## Data accessibility

This article has no additional data.
